# Supplementary material for: Hormone-induced mitochondrial fission is utilized by brown adipocytes as an amplification pathway for energy expenditure
Source: EMBO J. 2014 Jan 15;33(5):418–36. doi: 10.1002/embj.201385014 (PMC3983686; doi:10.1002/embj.201385014)
Supplement: Supplementary file 1 [file embj0033-0418-sd1.pdf]

THE
EMBO
JOURNAL

Manuscript EMBO-2013-85014

Hormone-induced mitochondrial fission is utilized by brown adipocytes as an amplification pathway for energy expenditure

Jakob D Wikstrom, Kiana Mahdaviani, Marc Liesa, Samuel B Sereda, Yaguang Si,Guy Las, Gilad Twig, Natasa Petrovic, Cristina Zingaretti, Adam Graham , Saverio Cinti, Barbara E Corkey, Barbara Cannon, Jan Nedergaard, and Orian S Shirihai

Corresponding author: Orian Shirihai, Boston University

Review timeline:

Submission date:	21 March 2013
Editorial Decision:	26 April 2013
Additional Author Correspondence:	26 April 2013
Addtional Editorial Correspondence:	29 April 2013
Revision received:	09 October 2013
Editorial Decision:	25 October 2013
Revision received:	19 November 2013
Accepted:	21 November 2013

Transaction Report:

(Note: With the exception of the correction of typographical or spelling errors that could be a source of ambiguity, letters and reports are not edited. The original formatting of letters and referee reports may not be reflected in this compilation.)

Editor: Thomas Schwarz-Romond

1st Editorial Decision 26 April 2013

Thank you very much for submitting your study that proposes mitochondrial fragmentation as potential amplification mechanism to regulate energy metabolism for consideration to The EMBO Journal editorial office.

I apologize for the delay in conveying a response caused by relatively late incoming assessments and a third scientist that originally agreed to review not having returned any comments. Please understand that I am therefore only able to reach a preliminary decision to not delay necessary further experimental requests.

I will be in touch as soon as I hear back from the third scientist, which could potentially influence the outcome of the current decision.

The attached reports indicate principle interest in your study. However, both scientists conclude that the paper would significantly benefit from definitive mechanisms linking NE/palmitate-stimulation

to the mitochondrial network dynamics and -uncoupling could be provided. Though not a principle requirement, ANY advance in this direction would be highly appreciated.

Both scientists outline additional controls and request further reaching experimentations. I would like to focus your attention specifically to the measurements of mitochondrial oxygen consumption and the inclusion of raw data for illustration (ref#1). Furthermore, expansions along the points 2, 4/5 and 7 of ref#2 would strengthen the interesting proposal of your study.

Based on amendments that constructively address these concerns, I am delighted to offer you the opportunity to revise the current dataset. Please do not hesitate to contact me with in case of further questions or to outline the timeframe for necessary experimental work (preferably via E-mail).

I hope that this outline of our referee's expectations facilitates efficient proceedings of necessary experimental improvements.

Please realize that I also have to remind you that the final decision on further proceedings at The EMBO Journal depends on the content of a thoroughly paper.

Lastly, I will be in touch in case I do hear back from the still outstanding referee.

REFEREE REPORTS:

Referee #1:

The manuscript is concerned with showing that NE is able to induce mitochondrial uncoupling in brown adipocytes by a mechanism that is not directly related to its actions in stimulating lipolysis. Namely, the authors suggest that NE induces a change in mitochondrial network architecture, and through this, stimulates UCP1-related thermogenesis.

Major comments:

Overall the microscopy data concerning the changes in mitochondrial network architecture are excellent. This section of the manuscript very thoroughly investigates the effects of NE and palmitate on mitochondrial architecture, the mechanism of fragmentation, and NE-induced changes in Opa1 cleavage and Drp1 activation. What seems to be missing is a direct indication of the pathway linking NE and the observed Opa1/Drp1 changes - the data skips directly to the effect on these proteins and the mitochondrial network. The paper would be strengthened with the addition of a possible mechanism.

The authors then go on to link this mitochondrial fragmentation with increased uncoupling, claiming this to be an essential step in NE-induced brown adipose thermogenic activation and the control of bioenergetic efficiency. This section needs additional work before the manuscript can be accepted. It appears that the authors have minimal experience in mitochondrial oxygen consumption measurements, and there are a number of major issues that need to be addressed before this manuscript can be accepted:
Firstly, the addition of antimycin A (or another ETC inhibitor) is necessary after any measurements of oxygen consumption, as a control to demonstrate that it is indeed mitochondrial ETC-based respiration that that is being measured.
Secondly, it is unclear whether the 'fold increase in oxygen consumption' is relative to cell number or protein content or simply per well of the seahorse plate. It is not labelled on the graph (or any of the other oxygen consumption graphs).
Thirdly, it would be informative to show the raw data in a concurrent graph alongside the 'fold increase in oxygen consumption' to avoid any ambiguity.
Fourth, figure 10(e) is unclear, and if possible should be explained more thoroughly or represented in a manner which is easier to understand. It is showing data 'normalised to scrambled control rates', but it is unclear as to which data this refers... If this is the data in section (d) reworked, then it should also show the effect of the MFN2 knockdown plus pamitate.

Fifth, in supplemental figure 5(b) and (c), it is unclear why the traces are dipping lower and reaching a new plateau at two timepoints. It looks as if there has been a seahorse injection, and while the legends indicate that this data represents NE or palmitate titration, it is not indicated what is occurring at those timepoints.

Overall, the microscopy and mitochondrial network data appears solid, and the respiration data can be salvaged, given some additional work. Additionally, the connection between NE/palmitate, mitochondrial network dynamics, and mitochondrial uncoupling is interesting, but would be strengthened with further explanation of the molecular mechanism involved. Lastly, the comment that NE is often used at maximal, supra-physiological levels (whereby its actions are completely different to those at physiological concentrations) is an excellent point, and very important to keep in mind when conducting experiments with hormones and other substances to investigate physiological processes.

Minor comments:

1. The authors quickly comment on the actions of an unsaturated fatty acid (oleate), showing that NE plus oleate induced more mitochondrial membrane depolarisation than NE plus palmitate. This is an interesting point that is only lightly touched upon, and warrants at the very least further discussion or commentary as to the potential mechanisms by which a difference in lipid species can affect BA uncoupling.

2. It would be optimal if the authors could include a measure of UCP1 activity, rather than just protein expression, since mitochondrial uncoupling is such a major part of the story.

3. Under the second heading of the discussion ('Mitochondrial fragmentation'), the authors mention that "activation of mitochondrial respiration is not associated with increased ATP synthesis". Firstly, the reference needs to be stated here (it is unclear if this is referring to one of the citations from the previous sentence). Secondly, a measure of ATP level would strengthen this conclusion, to indicate whether NE and palmitate can affect ATP production as well as mitochondrial membrane potential.

4. The authors show extremely clear mitochondrial swelling (figure 3e) upon treatment with NE and palmitate. Is it possible that the mitochondria are filled with lipids? A Nile red staining should be able to show this easily, if it can reach that level of sensitivity. It also appears as if the lipid droplets are smaller in the NE+palmitate cells - perhaps there is some lipolysis and movement of fatty acids into the mitochondrial matrix?

5. The supplementary figure legends are out of order. The authors deleted supplementary figure 1 but not the legend, and therefore all subsequent legends are referring to the incorrect figure.

Referee #2:

This paper describes a new role of mitochondrial dynamics in the metabolic transition from coupled to uncoupled respiration during thermogenesis in primary brown adipocytes. The authors conclude that norepinephrine (NE) and free fatty acids initiate uncoupling, and the decreased membrane potential leads to mitochondrial fragmentation, which in turn further induces uncoupling. Interestingly, uncoupling was partially inhibited by a dominant-negative form of Drp1 and stimulated by Mfn2 knockdown.

Major points

1. The oxygen consumption rates show a large variation among the figures. Even control cells have several fold differences, which are higher than the differences between control and experimental samples. Please explain these variations.
2. The authors suggest that the observed large spheroids by NE and palmitate are generated by fragmentation. However, given the large diameter and apparently no change in the number of

mitochondria per cell, these large spheroids may result from the loss of tubular morphology. What happens if cells are first treated with NE to induce fragmentation, and then with palmitate? Do mitochondria still form large spheroids? The authors should test if the number of mitochondria increased. Did the mitochondrial spheroids result from loss of cristae? It is necessary to analyze their ultrastructure using electron microscopy.
3. The authors suggest that uncoupling propagates from one side of the cell to the other, and this propagation is accompanied by mitochondrial fragmentation, but cells in Fig. 5A are already partially uncoupled, and the membrane potential shows a gradient. The membrane potential could become equally depolarized and maintain the original gradient. Unless the authors analyze cells that have a uniform membrane potential across their mitochondria before stimulation of uncoupling, the authors' model is not well supported. For the same reason, the image at 0 min should be included in Fig. 5B.
4. The authors indicated that NE plus palmitate treatment increases Drp1 puncta on mitochondria in Fig. 6A, but from the presented images, it is difficult to determine whether Drp1 is associated with mitochondria. What are the large puncta in the control? A better analysis is necessary.
5. Analysis of Drp1 is performed at 1 hr after treatment in Fig. 6. At this point, essentially all the membrane potential is lost. Therefore, the observed change may be the consequence of uncoupling. The same issue applies to Opa1 processing in Fig. 8. These experiments do not provide mechanisms for the regulation of mitochondrial dynamics in their current form.
6. The effect of dominant-negative Drp1 on mitochondrial shape is not clear in the images presented in Fig. 6. Higher magnification images would be needed to clearly show the hyperfused morphology.
7. Fig. 7 b and c show cells with mtPAGFP already losing their membrane potential at 0 min. Because the membrane potential is required for mitochondrial fusion, the effect of NE and palmitate on fusion cannot be addressed. Assuming that these pictures are likely representative images, the meaning of the quantification in Fig. 7d is unclear. The authors need to show changes in the value of the membrane potential (not relative values) in the same cells to validate their findings.

Minor points

1. A description of Fig. 2D is missing.
2. NE treatments cause fragmentation of mitochondria. Interestingly, the combination of NE and palmitate generates swollen mitochondrial spheres. Despite their striking morphological difference, the authors score both shapes as fragments in many figures. They should be quantitated separately.
3. The order of supplementary figures needs to be arranged properly. Some are mislabeled and missing.
4. It should be explained why MFN2 KD reaches higher maximum oxygen consumption rates than the control in Fig. 10. Is this because there are more cells that are depolarized in MFN2 KD? It would be helpful to include the quantitation.

Additional Author Correspondence 26 April 2013

Thank you very much for sending the decision letter. I very much appreciate the timely processing and the valuable guidelines for addressing the comments.

I am in Amsterdam for a conference where I presented this story in a talk. I got very positive feedback with a number of scientists pointing to evidence they collected in various systems that support our new concept of mitochondrial fragmentation as a mechanism that facilitate uncoupling and increased energy expenditure.

During the past month we generated some significant new data that makes this story stronger and more exciting.

I am looking forward to send you a revised version.

Addtional Editorial Correspondence 29 April 2013

Thank you very much for sharing this with me.

I did in the meantime receive final comments from the third referee. As before, this scientist recognizes the potential interest in your intriguing proposal, remains however unconvinced at this stage, particularly contesting that mitochondrial fragmentation would explain enhanced mitochondrial function. S/he further suggests to run a comparison with similar analyses in white fat. With this rather preliminary state of affairs, ref#3 is the most critical, currently recommending publication in a more specialized venue (detailed comments below).

Based on your recent correspondence that indicates existence of further reaching experimentation, I still maintain our invitation to submit a revised version, urge you however to attend to this additional remarks as best as you can!

REFEREE REPORT:

Remarks to the Author:

There are several critical issues that make this manuscript preliminary for publication. The data analyses of multiple experiments are incomplete and/or inappropriate and not easy to interpret:

1. The ref: Molina et al 2009b is actually not in the list.
2. For Fig. 1B-C no explanation is provided for the synergistic effect.
3. The analyses and interpretation of the effects of palmitate versus oleate is incomplete.
4. Suppl. Fig. 1D does not exist.
5. Data for fission events splitting into two daughter mitochondria should be shown.
6. Is Drp1 DN - dominant negative?
7. The issue regarding mitochondrial fragmentation being associated with enhanced mitochondrial function is controversial and unlikely to explain the authors' observation.
8. Not clear if Fig 2b refers to N+P from previous experiment.
9. The fold change in Fig 2 c appears different from the data in Fig 1b,c.
10. Fig 3. It is important to show the comparison with white fat.
11. In Fig 7, it is unclear why there is no synergistic effect of NE and P.
12. Suppl. Fig 4b: no normalization protein is shown for UCP1 blot!!
13. Suppl. Fig 6: upper third panel is not labeled how many hours post recovery.
14. The Discussion is extremely long and reads like a thesis.

1st Revision - authors' response 09 October 2013

Referee #1:

The manuscript is concerned with showing that NE is able to induce mitochondrial uncoupling in brown adipocytes by a mechanism that is not directly related to its actions in stimulating lipolysis. Namely, the authors suggest that NE induces a change in mitochondrial network architecture, and through this, stimulates UCP1-related thermogenesis.

Major comments:

1) Overall the microscopy data concerning the changes in mitochondrial network architecture are excellent. This section of the manuscript very thoroughly investigates the effects of NE and palmitate on mitochondrial architecture, the mechanism of fragmentation, and NE-induced changes in Opa1 cleavage and Drp1 activation. What seems to be missing is a direct indication of the pathway linking NE and the observed Opa1/Drp1 changes - the data skips directly to the effect on these proteins and the mitochondrial network. The paper would be strengthened with the addition of a possible mechanism.

We thank this reviewer for stating that our imaging data is excellent and for his/her insightful comments regarding the mechanism of fragmentation. We have provided two new sets of experiments providing a mechanism for fragmentation:

a) We now provide new data identifying the mechanism by which NE activates Drp1-mediated fragmentation. In the previous version of the manuscript, we showed that Drp1 was phosphorylated and formed puncta, which is characteristic of increased Drp1-mediated fission (our data, Han et al, JCB and others). We now show that Drp1 Ser600 phosphorylation stimulated by NE requires Protein Kinase A (PKA) activity. PKA has been widely described to be activated by NE through an increase in cAMP levels, caused by G protein-coupled β –adrenergic receptors (β-ARs). This adrenergic pathway is also known as the canonical pathway for brown fat stimulation (Cannon and Nedergaard, 2004). We now show that inhibition of PKA by treatment with the established PKA inhibitor H89 prevented the increase in Drp1 phosphorylation and mitochondrial fragmentation induced by NE. Thus, we identified the mechanism by which the canonical pathway of BAT stimulation increases Drp1-mediated fission. These results are shown in Figure 6.

b) In this context, another question to be addressed was whether mitochondrial depolarization *per se* was the main activator of Drp1-mediated fission (as shown in Cereghetti et al., 2008) and not Drp1 phosphorylation. In order to address this, we inhibited lipolysis in BA treated with NE using Orlistat. Inhibition of lipolysis prevents the release of FFA from lipid droplets, which activate UCP1 and thus mitochondrial depolarization. New Figure 7 shows that treatment with Orlistat prevented mitochondrial depolarization after NE, but not mitochondrial

fragmentation in primary BA. This latter result demonstrates that NE induces Drp1-mediated mitochondrial fission independently of mitochondrial depolarization. Moreover, Orlistat treatment prevented Opa1 cleavage, demonstrating that mitochondrial depolarization induced by lipolysis and FFA release is the mechanism for Opa1 degradation and likely decreased mitochondrial fusion rates.

2) The authors then go on to link this mitochondrial fragmentation with increased uncoupling, claiming this to be an essential step in NE-induced brown adipose thermogenic activation and the control of bioenergetic efficiency. This section needs additional work before the manuscript can be accepted. It appears that the authors have minimal experience in mitochondrial oxygen consumption measurements, and there are a number of major issues that need to be addressed before this manuscript can be accepted:

2a) Firstly, the addition of antimycin A (or another ETC inhibitor) is necessary after any measurements of oxygen consumption, as a control to demonstrate that it is indeed mitochondrial ETC-based respiration that is being measured.

We thank the reviewer for this important point. Indeed, Antimycin A was added in our experiments (third Seahorse injection, port C) and we consistently detected a large decrease in respiration after its addition. In this regard, we already showed Antimycin A injections in the first version of this manuscript in Supplementary Figures 5b and 5c of the previous version. However, the injections labels in these Supplementary Figures were missing (see following point 2e). We apologize for this formatting error that lead to this comment from the reviewer. We thank the reviewer for highlighting this issue. As shown in current Supplementary Figure 1, respiration values in BA under Antimycin A were ~50 pmols O_2/min and thus close to background rates. Following the reviewers indication, we now also show Antimycin A injections in the main Figures of the manuscript (Figure 1, 8, 9 and supplemental 1 and 6).

We also want to state that our laboratory does not have minimal experience performing respirometry. Shirihai laboratory, developed the first high throughput protocol using the Seahorse XF24 to perform respirometry in intact pancreatic islets (Wikstrom et al., 2012 PLoS One). Moreover, Shirihai laboratory was the first to publish the use of isolated mitochondria using the Seahorse XF24 (Sahin et al., Nature 2011), a protocol that was also developed in our laboratory together with Seahorse Biosciences. Shirihai laboratory had one of the first ten XF24 analyzers to be installed outside Seahorse and performed oxygen consumption measurements in intact cells about 7 years ago, comparing them to the Clark electrode. These results were presented in our previous publication (Twig et al., EMBO J. 2008). Finally, as an objective measure of our experience in respirometry, our studies using the XF24 have been peer-reviewed in multiple

journals and are presented in more than 15 publications. Co-authors Nedergaard and Cannon lab have participated in the design of respirometry experiments and analyses of this data. Drs. Nedergaard and Cannon have over 30 years of experience in BAT bioenergetics.

***2b) Secondly, it is unclear whether the 'fold increase in oxygen consumption' is relative to cell number or protein content or simply per well of the seahorse plate. It is not labeled on the graph (or any of the other oxygen consumption graphs)*.**

In Figure 2 (and Figure 1 in new version), the fold increase in oxygen consumption represents the increase in brown adipocyte oxygen consumption induced by the acute injection (within the Seahorse ports) of NE, Palmitate, Oleate or their combination with NE. This fold increase is calculated by the maximal values of oxygen consumption rates (OCR pmols O_2/min) after NE, Oleate, Palmitate injection divided by the absolute values of OCR before the addition of these compounds and multiplied by 100 (percentage).

Given that these effects are measured before and after a treatment in the same BA and the same initial number of cells was used in all treatments (see material and methods), no correction per cell number or protein content is required. In this regard, the ”fold increase in oxygen consumption” shown represents the average of multiple independent experiments (up to 13 independent experiments) as stated in the Figure 1 legend. Thus, the bar graphs do not represent the average of replicates/wells of the same experiment.

2c) Thirdly, it would be informative to show the raw data in a concurrent graph alongside the 'fold increase in oxygen consumption' to avoid any ambiguity.

We followed the reviewer indication and we now show raw data traces from a representative experiment and their replicates in Figure 1, 8 and 9 and supplemental 1 and 6. These data are now shown alongside the “fold increase in oxygen consumption”, which average multiple experiments.

Of note, primary BA respiration assays can show variability in absolute respiration values (pmols O_2/min) between independent experiments in some cases. This variability is the rationale behind presenting the data as fold increase in oxygen consumption in some cases. The main cause of this variability is the actual number of differentiated BA obtained per Seahorse plate in each independent experiment (isolation from mice). As noted in the materials and methods, we isolated primary pre-adipocytes from brown adipose tissue, we then plated the same number of cells in all the wells of a Seahorse plate and differentiated them to BA within the plate. These cells cannot be lifted from the plate and re-plated at a new density. This generate variability between experiments and require that each experiment will have its own controls, then calibrated. It is important to note that undifferentiated preadipocytes do not increase respiration in response to NE and have much lower basal respiration rates (as their mitochondrial content is very low when compared to differentiated

BA). As a consequence, there are 2 major components affecting the number of fully differentiated BA within a Seahorse plate in some independent experiments: the viability of pre-adipocytes within the preparation of each independent experiment and their differentiation efficiency. Therefore, the changes in respiration over control/untreated BA (i.e. before treatment, control virus…) are always more consistent and reproducible between independent experiments than the average of absolute oxygen consumption rates. This is the reason why all treatments are performed in the same experiment/isolation of primary BA. Then, in the cases where variability is high, the fold change in respiration over control/untreated BA of each independent experiment is averaged and shown in bar graphs ± SEM. In the cases in which variability is not high, the average of raw data of independent experiments is shown.

2d) Fourth, figure 10(e) is unclear, and if possible should be explained more thoroughly or represented in a manner which is easier to understand. It is showing data 'normalized to scrambled control rates', but it is unclear as to which data this refers... If this is the data in section (d) reworked, then it should also show the effect of the MFN2 knockdown plus palmitate.

We apologize for the lack of clarity. The reviewer is correct that previous Figure 10E (Figure 9 in the new version) was the data in section (d) reworked. Old Figure 10 has been completely redone as we have triplicated the amount of data. These include new experiments with Mfn2 KD and with additional concentrations of oleate and palmitate (Figure 9 in the current version). We show the actual raw traces of representative experiments with replicates. In addition, we have provided a new bar graph from the traces in a manner that it is easier to understand, by grouping control (scrambled) vs. Mfn2 KD as pairs of bar graphs, pairing them according to the experimental groups (NE, palmitate…). See new Figure 9 for details. We have also clarified the Figure legend.

2e)Fifth, in supplemental figure 5(b) and (c), it is unclear why the traces are dipping lower and reaching a new plateau at two timepoints. It looks as if there has been a seahorse injection, and while the legends indicate that this data represents NE or palmitate titration, it is not indicated what is occurring at those timepoints.

We have clarified this and we apologize for the missing injection labels, which are directly related to the previous point 2a mentioned by the reviewer. Supplemental Figures 5C is now part of supplemental Figure1b. The reviewer is right that NE and palmitate titration are performed through a Seahorse injection in port A (first increase in respiration), followed by an injection of oligomycin in port B (first decrease in respiration) and then by an injection of Antimycin A in port C (second decrease in respiration). We have added these labels.

3) Overall, the microscopy and mitochondrial network data appears solid, and the respiration data can be salvaged, given some additional work. Additionally, the connection between NE/palmitate, mitochondrial network dynamics, and mitochondrial uncoupling is interesting, but would be strengthened with further explanation of the molecular mechanism involved.
We have provided an hypothesis in the discussion and a new model, showing that fragmentation leading to swelling might be remodeling cristae structure, decreasing ATP synthase activity as a consequence (through changes in the curvature of the inner membrane) and causing a better exposure of UCP1 to FFA released by lipolysis.

4) Lastly, the comment that NE is often used at maximal, supra-physiological levels (whereby its actions are completely different to those at physiological concentrations) is an excellent point, and very important to keep in mind when conducting experiments with hormones and other substances to investigate physiological processes.

We are grateful to the reviewer for this comment. Indeed, the use of this lower concentration of NE is the experiment that revealed that NE is sensitizing the brown adipocyte to their activation by exogenous FFA. This is further emphasized in the results section and the discussion.

Minor comments:

1. The authors quickly comment on the actions of an unsaturated fatty acid (oleate), showing that NE plus oleate induced more mitochondrial membrane depolarisation than NE plus palmitate. This is an interesting point that is only lightly touched upon, and warrants at the very least further discussion or commentary as to the potential mechanisms by which a difference in lipid species can affect BA uncoupling.

The mechanism by which FFA activate UCP1 has been recently reported (Fedorenko et al., Cell 2013). This study thoroughly discusses and demonstrates that different lipid species show different capacity to activate UCP1. One of the main findings is that the longer the hydrophobic tail of the FFA is, the more efficiently it activates UCP1 (Fedorenko et al., Cell 2013). Oleate has 18 carbons and an insaturation, whereas palmitate is saturated and has 16 carbons. Therefore, although they were not directly compared in the cited study, oleate could be a better activator of UCP1 as it has a longer hydrophobic tail. This commentary has been added to the manuscript.

2. It would be optimal if the authors could include a measure of UCP1

activity, rather than just protein expression, since mitochondrial uncoupling is such a major part of the story.

The only established methodologies to measure UCP1 activity are patch clamp of submitochondrial particles (Fedorenko et al., 2013, Cell) or State 4 respiration inhibition by GDP and activated by FFA in isolated mitochondria. The main problem is that both approaches require cellular disruption and thus elimination of mitochondrial dynamics (fusion and fission) and in vivo morphology. Therefore, with the current methodologies available, it is not feasible to specifically measure UCP1 activity without altering normal mitochondrial dynamics.

3. Under the second heading of the discussion ('Mitochondrial fragmentation'), the authors mention that "activation of mitochondrial respiration is not associated with increased ATP synthesis". Firstly, the reference needs to be stated here (it is unclear if this is referring to one of the citations from the previous sentence). Secondly, a measure of ATP level would strengthen this conclusion, to indicate whether NE and palmitate can affect ATP production as well as mitochondrial membrane potential.

We have reviewed the current evidence linking this in a review article published in Cell Metabolism (Liesa and Shirihai 2013). As a summary, the uncoupler FCCP can increase respiration 4 fold (and thus increase mitochondrial electron transport chain activity 4 fold) and inhibit mitochondrial ATP synthesis. FCCP has a similar effect than UCP1, which is the basis of the thermogenic of UCP1 and mitochondrial uncouplers.
Measuring cellular ATP levels could be confusing, as not only mitochondria can contribute to total ATP levels (i.e. glycolysis to lactate). Thus, mitochondrial uncoupled respiration could be compensated by other pathways and it would not be reflected in any change in total ATP levels.

4. The authors show extremely clear mitochondrial swelling (figure 3e) upon treatment with NE and palmitate. Is it possible that the mitochondria are filled with lipids? A Nile red staining should be able to show this easily, if it can reach that level of sensitivity.
We tried this experiment by using bodipy labeled FFA. We preferred the bodipy-FFA as we did not expect that large lipid droplets (mostly constituted by triacylglicerides and stained by Nile Red) would be able to enter inside the mitochondria. In addition, such accumulation of neutral lipids
However, we failed to detect any bodipy signal inside the swelled mitochondria, suggesting that bodipy-FFA are not imported into the mitochondria or are just beta-oxidized, releasing the bodipy out in the cytosol.

4a) It also appears as if the lipid droplets are smaller in the NE+palmitate cells - perhaps there is some lipolysis and movement of fatty acids into the mitochondrial matrix? The reviewer is right and FFAs from lipolysis are the

main activators of UCP1 and mitochondrial depolarization. Indeed, our results using the lipolysis inhibitor Orlistat, which prevents mitochondrial depolarization but not fragmentation, confirms the reviewer's point.

5. The supplementary figure legends are out of order. The authors deleted supplementary figure 1 but not the legend, and therefore all subsequent legends are referring to the incorrect figure.

This has been corrected.

Referee #2:

This paper describes a new role of mitochondrial dynamics in the metabolic transition from coupled to uncoupled respiration during thermogenesis in primary brown adipocytes. The authors conclude that norepinephrine (NE) and free fatty acids initiate uncoupling, and the decreased membrane potential leads to mitochondrial fragmentation, which in turn further induces uncoupling. Interestingly, uncoupling was partially inhibited by a dominant-negative form of Drp1 and stimulated by Mfn2 knockdown.

Major points
1. The oxygen consumption rates show a large variation among the figures. Even control cells have several fold differences, which are higher than the differences between control and experimental samples. Please explain these variations.

We thank the reviewer for this comment. As discussed in the remarks of reviewer 1, the main cause of this variability is the actual number of differentiated BA obtained per Seahorse plate in each independent experiment (isolated from BAT of different mice). As noted in the materials and methods, we isolated primary pre-adipocytes from brown adipose tissue, we then plated the same number of cells in all the wells of a Seahorse plate and then differentiated them to BA within the plate. It is important to note that undifferentiated preadipocytes do not increase respiration in response to NE and they have much lower basal respiration rates (as their mitochondrial content is very low when compared to differentiated BA). As a consequence, there are 2 major components affecting the absolute respiration rates in some independent experiments: the viability of pre-adipocytes within the preparation of each independent experiment and their differentiation efficiency. Therefore, the changes in respiration over control/untreated BA (i.e. before treatment, control virus…) are always more consistent and reproducible between independent experiments than the average of absolute oxygen consumption rates.

This is the reason why all the treatments are performed in the same experiment/isolation of primary BA. Then, in the cases where variability is high,

the fold change in respiration over control/untreated BA of each independent experiment is averaged and shown in bar graphs ± SEM. In the cases in which variability is not high for the same experimental series, the average of raw data of independent experiments is shown.

2. The authors suggest that the observed large spheroids by NE and palmitate are generated by fragmentation. However, given the large diameter and apparently no change in the number of mitochondria per cell, these large spheroids may result from the loss of tubular morphology. What happens if cells are first treated with NE to induce fragmentation, and then with palmitate? Do mitochondria still form large spheroids? The authors should test if the number of mitochondria increased. Did the mitochondrial spheroids result from loss of cristae? It is necessary to analyze their ultrastructure using electron microscopy.

We now dedicate 8 panels (Figure 3A-G and Figure 2h) to the characterization of the spheroids and provide new data that validates the large spheroid formation in vivo in BAT from cold exposed mice, suggesting that physiological adrenergic (NE) stimulation induced by cold exposure is sufficient to induce spheroids (TOM20 IHC, FIGURE 2h). We also provide TEM and SEM images of the enlarged mitochondria from BAT and cultured BA. Concerning the cristae structure, we show that BAT mitochondria from cold exposed mice or treated with NE are swelled but maintained cristae structure (Figure 3e and g and supplemental Figure 2a). This is in agreement to previous studies (Desautels & Himms-Hagen, 1980) (Vallin, 1970).

3. The authors suggest that uncoupling propagates from one side of the cell to the other, and this propagation is accompanied by mitochondrial fragmentation, but cells in Fig. 5A are already partially uncoupled, and the membrane potential shows a gradient. The membrane potential could become equally depolarized and maintain the original gradient. Unless the authors analyze cells that have a uniform membrane potential across their mitochondria before stimulation of uncoupling, the authors' model is not well supported. For the same reason, the image at 0 min should be included in Fig. 5B.

We thank the reviewer for this valid point. First, we would like to point that unless stimulated with NE or NE+P BA do not show a gradient in membrane potential. In the new version we present multiple images of BA that were not stimulated with NE. These cells do not show a gradient in membrane potential (Figures 1 and 2).

Second, we agree with the reviewer main point about the labeling of time in figure 5A of the previous version. The label “time 0” refers to the first image obtained after addition of NE, which is timed to less than 1 minute post addition. We agree that labeling it “Time 0” could be misleading and we have corrected this. The reason for the gradient in what we called “time 0” image was the delay between the addition of NE and the image capture, as confocal imaging could not

be performed during the application and mixing of NE in the dish. The existence of the gradient is caused by the rapid nature of BA activation by NE. In addition, we show new data that inhibition of lipolysis by Orlistat prevents mitochondrial depolarization induced by NE. BAs under Orlistat do not show any gradient, further confirming the absence of a gradient without stimulation.

4. The authors indicated that NE plus palmitate treatment increases Drp1 puncta on mitochondria in Fig. 6A, but from the presented images, it is difficult to determine whether Drp1 is associated with mitochondria. What are the large puncta in the control? A better analysis is necessary.

We agree with the referee. In the revised version of the manuscript we added co-localization experiments. In the revised version of Figure 6 we included images of Drp1 co-localizing with mitochondria (Figure 6A).

5. Analysis of Drp1 is performed at 1 hr after treatment in Fig. 6. At this point, essentially all the membrane potential is lost. Therefore, the observed change may be the consequence of uncoupling. The same issue applies to Opa1 processing in Fig. 8. These experiments do not provide mechanisms for the regulation of mitochondrial dynamics in their current form.

We have performed 2 new sets of experiments showing that NE can mediate fragmentation in the absence of mitochondrial depolarization and that Drp1 is phosphorylated in a PKA dependent manner. We prevented mitochondrial depolarization by treating BA with a lipolysis inhibitor (Orlistat), which prevents FFA release and thus UCP1 activation. NE caused Drp1 phosphorylation and mitochondrial fragmentation in BA treated Orlistat. However, Opa1 cleavage did not occur in BA. Thus, altogether these experiments that NE activation of PKA increases Drp1 phosphorylation and fission independently of depolarization, whereas inhibition of fusion by Opa1 cleavage is dependent on FFA mediated depolarization.

6. The effect of dominant-negative Drp1 on mitochondrial shape is not clear in the images presented in Fig. 6. Higher magnification images would be needed to clearly show the hyperfused morphology.

We thank the reviewer for the comment. In the revised version of the figure (Figure 8 in the revised manuscript) higher magnification images are shown, illustrating the characteristic ball and thread mitochondrial structures of Drp1 DN overexpression.

7. Fig. 7 b and c show cells with mtPAGFP already losing their membrane potential at 0 min. Because the membrane potential is required for mitochondrial fusion, the effect of NE and palmitate on fusion cannot be addressed. Assuming that these pictures are likely representative images,

the meaning of the quantification in Fig. 7d is unclear. The authors need to show changes in the value of the membrane potential (not relative values) in the same cells to validate their findings.

Figure 7a, b and c (Figure 5 a-d in the new version) show the quantification of mitochondrial fusion rates **after** the treatment with NE, NE+palmitate, palmitate alone or control. This means that PAGFP was photoactivated **after** the treatment occurred (and thus after **physiological** depolarization occurred). Time 0 labeled in these figures is Time 0 after photoactivation of GFP, not time 0 after treatment. Time 0 for photoactivation is time 30 min for NE treatment. This is also the reason for the green swollen mitochondria seen at time 0 of photoactivation. We added a clarification of the time terminology in the legends of Figure 5.

The quantification in Figure 7c (figure 5d in the new version) shows that only BA treated for 1 hour with NE or NE+palmitate show inhibition of fusion rates. This result demonstrates that NE-mediated fragmentation and depolarization in activated BA is not only associated with increased fission, but also with decreased fusion. This is the first report showing that mitochondrial fusion is inhibited in activated BA and Figure 7c represents the quantification of mitochondrial fusion rates, measured as the average fluorescence intensity decay with time. This is the first evidence that physiological depolarization (by UCP1 activation) inhibits mitochondrial fusion. While this was somehow expected because non-physiological FCCP or depolarization treatments were shown to inhibit fusion, there was no evidence to date that this occurred in the physiological context of UCP1 activation. Therefore, this Figure is informative and relevant to understand brown fat mitochondrial physiology.

Minor points

1. A description of Fig. 2D is missing.

This has been included in the revised version (Supplementary figure 5).

2. NE treatments cause fragmentation of mitochondria. Interestingly, the combination of NE and palmitate generates swollen mitochondrial spheres. Despite their striking morphological difference, the authors score both shapes as fragments in many figures. They should be quantitated separately.

We have provided these quantifications in Figure 3f and g. In the case of NE+P a 60% of cells show fragmentation and 60% of these same cells show swelling. This means that a 100% of cells showing fragmentation have swelled mitochondria. On the other hand, close to 55% of BA under NE show fragmentation, but only a 5% of the total cells show swelling, meaning that more than 10% of the cells under NE with fragmentation show swelling. This has been clarified in the text.

3. The order of supplementary figures needs to be arranged properly. Some are mislabeled and missing.
This has been corrected.

4. It should be explained why MFN2 KD reaches higher maximum oxygen consumption rates than the control in Fig. 10. Is this because there are more cells that are depolarized in MFN2 KD? It would be helpful to include the quantitation.

Since we find that the fold increase in oxygen consumption is higher in Mfn2 KD, we rule out the possibility that this is due to increase in the number of total cells in the Mfn2 KD culture (Figure 9). We quantified UCP1 expression and did not find a difference between Mfn2 KD and WT indicating that there was no difference in the number of differentiated cells. We quantified NE-induced lipolysis and found no significant difference between Mfn2 and control (and a trend toward reduced lipolysis in Mfn2 KD). This is suggesting that there is no difference in the number of responding cells. This is shown in Figure 9F. We could not find a difference in NE-induced depolarization when comparing Mfn2 KD to control BA (data not shown). These results show that there was the same amount of depolarized cells in Mfn2 KD.

Referee#3

There are several critical issues that make this manuscript preliminary for publication. The data analyses of multiple experiments are incomplete and/or inappropriate and not easy to interpret:

We thank the reviewer for the valuable comments and suggestions. The revised version has a number of new experiments including experiments in vivo and experiments that shed light on the mechanism of the observed changes.

1. The ref: Molina et al 2009b is actually not in the list.
This has been corrected and the reference was updated to Molina et al., 2009.

2. For Fig. 1B-C no explanation is provided for the synergistic effect.
The results section for the synergistic effect has been rewritten and an explanation of the synergistic effect is provided. We now discuss and illustrate a suggested model by which NE contributes both the induction of lipolysis as well as mitochondrial fragmentation. Mitochondrial fragmentation facilitates the capacity of FFA to induce uncoupling. We demonstrate that by knocking down the fusion protein, Mfn2, we can elicit a significantly larger uncoupling response in BA exposed to a blood levels of fatty acids, in the absence of adrenergic stimulation.

3. The analyses and interpretation of the effects of palmitate versus oleate is incomplete.

We added new experiments that compare the effects of oleate to palmitate in terms of synergistic effect with NE (Figure1), and their interaction with Mfn2 KD cells (Figure 9).
A new paragraph concerning the analysis and interpretation of the differences has been added to the discussion. We explain that differences are likely explained by the recent report showing how FFA activate UCP1.

4. Suppl. Fig. 1D does not exist.
We apologize for this error. This has been corrected and the reference to the correct figure is now provided.

5. Data for fission events splitting into two daughter mitochondria should be shown.

We have removed the sentence describing the frequency of fission events as the current methodologies do not provide an accurate enough measure of fission, mostly due to the fact that more that 95% of touching events do not proceed to fusion. When a touching event ends, it may be counted as a fission event.

However, we provide new data that shows that NE induces DRP1 phosphorylation and the colocalization of Drp1 with mitochondria, supporting the induction of fission.

6. Is Drp1 DN - dominant negative?

We apologize for the lack of clarity. This is correct. We have now included in the text "Drp1 Dominant Negative (Drp1 DN)" for clarification.

7. The issue regarding mitochondrial fragmentation being associated with enhanced mitochondrial function is controversial and unlikely to explain the authors' observation.

We thank the reviewer for bringing up this point. Fragmentation has been thought to be associated only with cell death and pre-death stress. However, this view is changing and replaced with the understanding that fragmentation is an adaptive response. This has been recently described in a review in Cell Metabolism (Liesa and Shirihai, 2013) a summary of multiple studies showing increased respiratory activity in conditions associated with mitochondrial fragmentation are reviewed. One of the examples is treatment with the uncoupler FCCP. FCCP is used to uncouple mitochondrial respiration (in a similar manner as UCP1) and it is used to measure maximal respiratory capacity. FCCP also induces mitochondrial

fragmentation.

8. Not clear if Fig 2b refers to N+P from previous experiment.
In the revised version we have merged figure 1 and 2, thus clarifying this issue.

9. The fold change in Fig 2 c appears different from the data in Fig 1b,c.

Yes, indeed these differ as they report on different parameters. Figure 2c (merged with Figure 1 in the new version) quantifies the % of cells that depolarized (showed a >30% decrease in TMRE fluorescence intensity) as a% of the total number of cells. Figure 1c quantifies the average depolarization in mV. Fig 1b show representative images from the treatments used to obtain Figure 1a and Figure 1e.

10. Fig 3. It is important to show the comparison with white fat.
We thank the referee for the suggestion. However, this will draw from the focus of this study which examines the role of changes in mitochondrial architecture during stimulation of thermogenesis through uncoupling. This is a process that is unique to brown adipocytes. Thermogenic and uncoupling activity are by orders of magnitude higher in BAT than white fat. Comparison to white adipocytes would not add to our understanding of the brown adipocyte thermogenesis. When considering thermogenesis, muscle is closer to brown adipocyte, as compared to white adipocytes. This manuscript describes architectural changes and their contribution to thermogenesis in brown adipocytes. We suggest that comparison to other tissues, including white adipocytes belong in followup papers.

We would also like to add that we have examined changes in mitochondrial architecture in white adipocytes. When comparing white to brown adipocytes the regulation and impact of mitochondrial fragmentation show some similarities along with multiple differences. Based on what we found thus far, the biology of mitochondrial architecture in white adipocytes deserves its own paper focused on white adipocytes.

11. In Fig 7, it is unclear why there is no synergistic effect of NE and P.
Because the depolarization induced by NE is sufficient to inhibit mitochondrial fusion rates to their minimum.

12. Suppl. Fig 4b: no normalization protein is shown for UCP1 blot!!

We thank the reviewer for comment. In the revised version this has been addressed and a loading control is included.

13. Suppl. Fig 6: upper third panel is not labeled how many hours post recovery.

We thank the referee for pointing this error. This has been addressed in the revised version, where this panel appears in Figure 4f. In the revised version we indicate that this image was taken 5 minutes after washing of NE and replacing of the media.

14. The Discussion is extremely long and reads like a thesis.

The discussion has been re-written and the content was decreased a 50%.

2nd Editorial Decision 25 October 2013

Thank you very much for the revised study.

One of the original referees assessed the revised paper, supporting publication without further amendments.

Before formal acceptance though, please notice that The EMBO Journal encourages the publication of source data, particularly for electrophoretic gels/blots, with the aim to make primary data more accessible and transparent to the reader. This entails presentation of un-cropped/unprocessed scans for KEY data of published work. We would be grateful for one PDF-file per figure with such information. These will be linked online as supplementary "Source Data" files.

Please allow me to congratulate you to your study. I look forward to receiving relevant source data and confirm that the editorial office will be in touch soon with necessary paperwork related to official acceptance.
