# Supplementary figures and images for: Hormone-induced mitochondrial fission is utilized by brown adipocytes as an amplification pathway for energy expenditure
Source: EMBO J. 2014 Jan 15;33(5):418–36. doi: 10.1002/embj.201385014 (PMC3983686; doi:10.1002/embj.201385014)

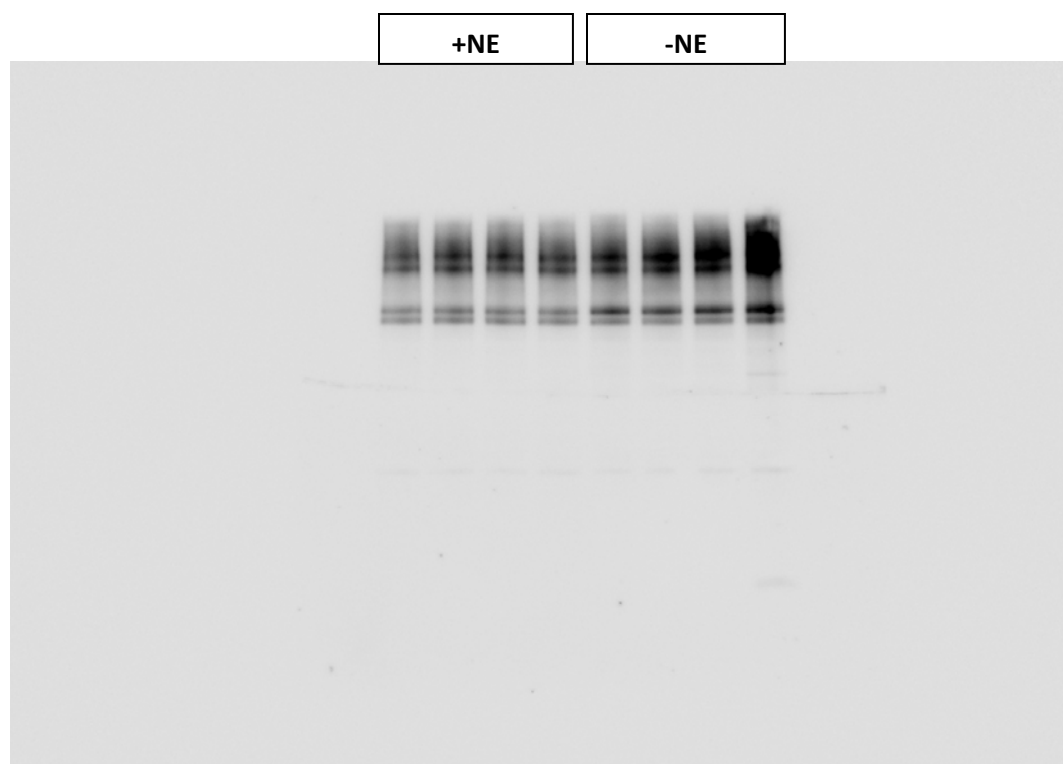

The original WB of the **Opa1** +/- NE

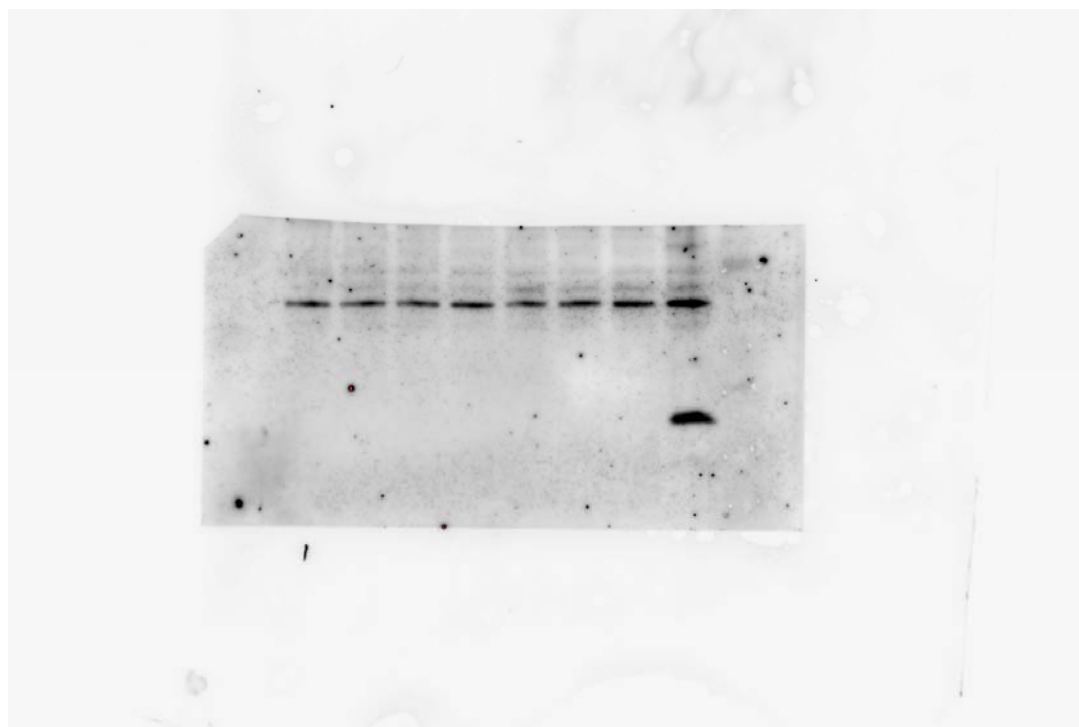

The original WB of the **Porin** +/- NE

Supplement: Supplementary file 2 [file embj0033-0418-sd2.pdf]

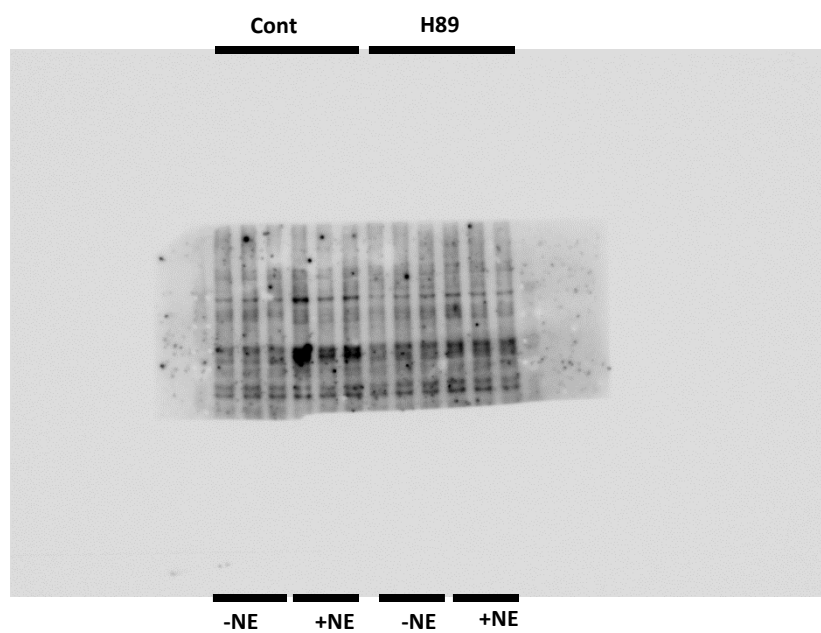

The original WB of the **p-Drp1(Ser600)** +/- NE and +/- H89

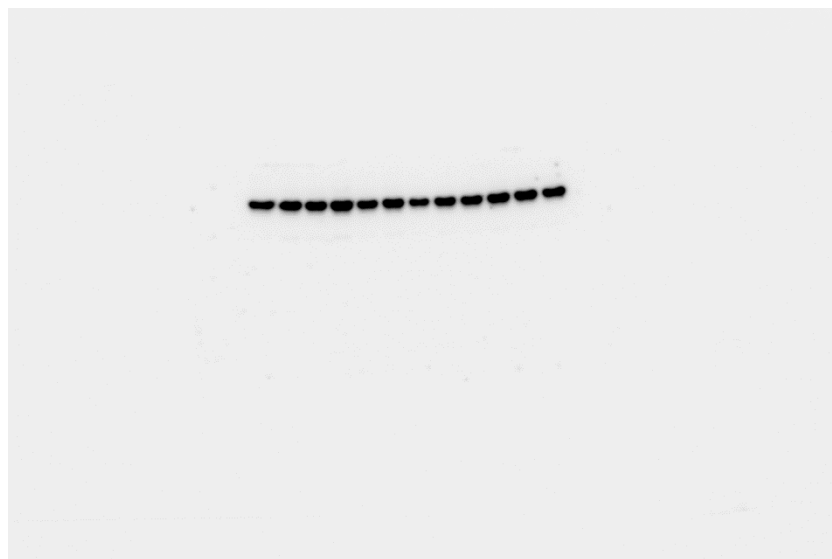

The original WB of the **Porin** +/- NE and +/- H89

Supplement: Supplementary file 3 [file embj0033-0418-sd3.pdf]

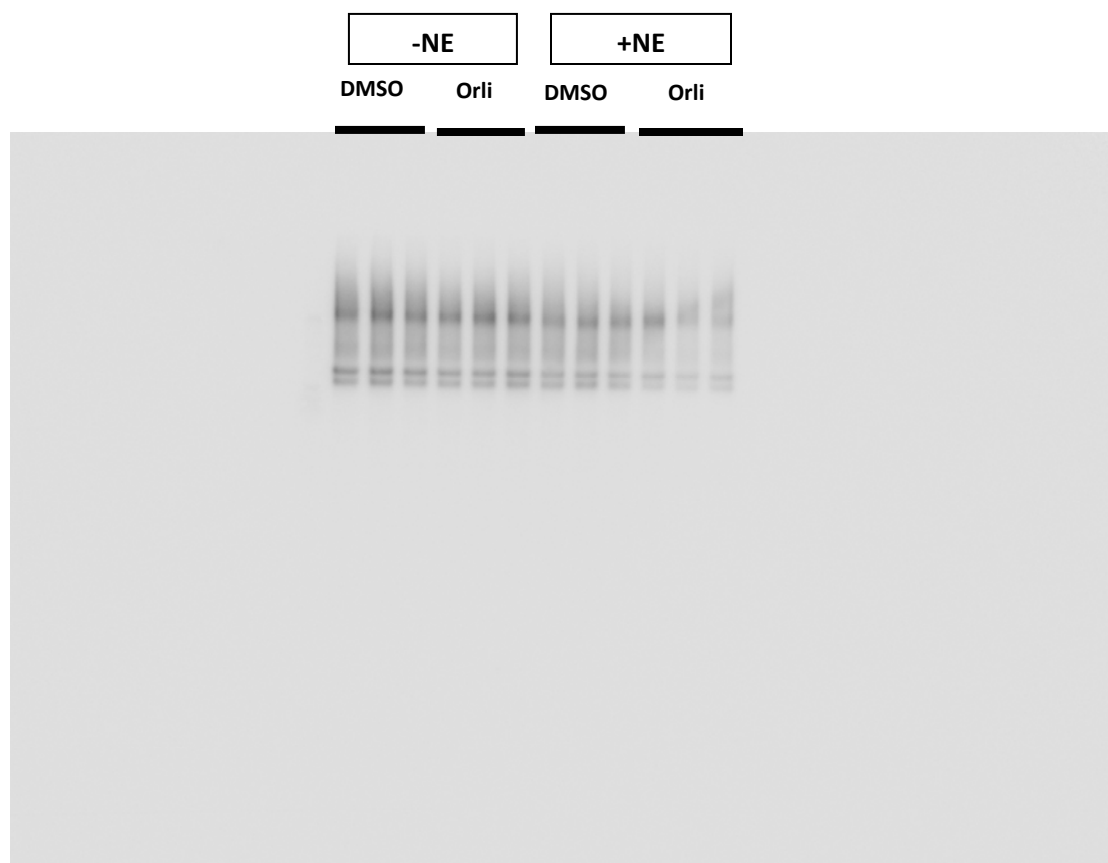

The original WB of the **Opa1** +/- NE and +/- orlistat

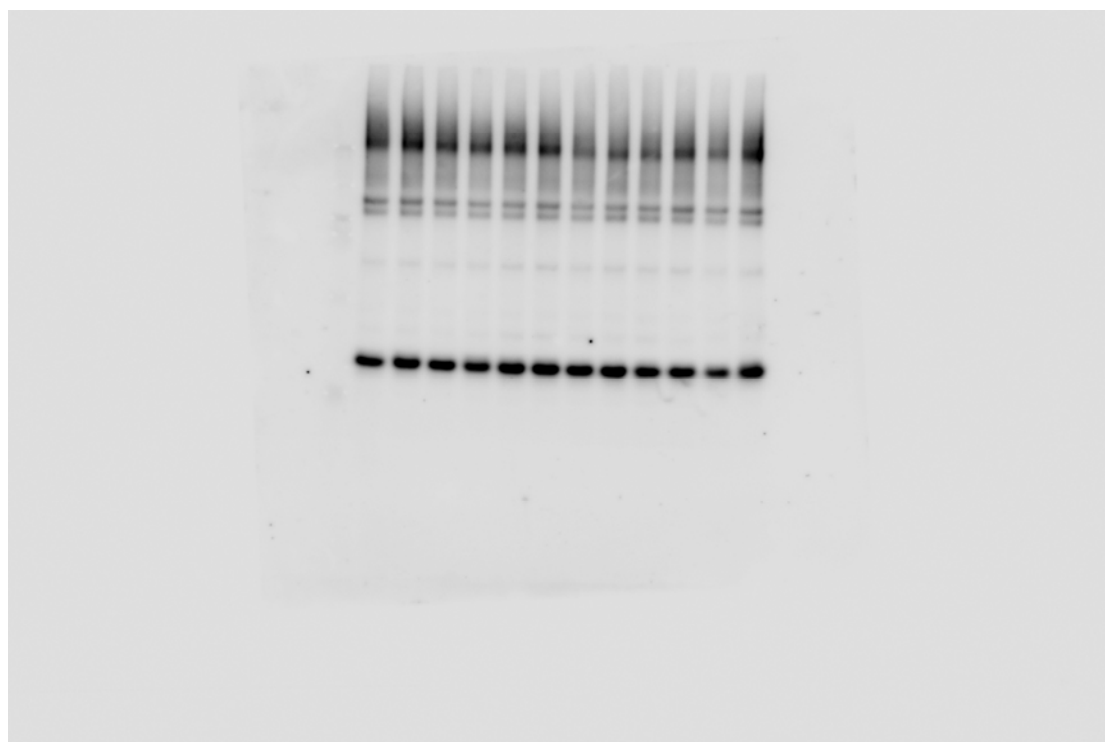

The original WB of the **Porin** +/- NE and +/- Orlistat

Supplement: Supplementary file 4 [file embj0033-0418-sd4.pdf]
