# Supplementary material for: Hormone-induced mitochondrial fission is utilized by brown adipocytes as an amplification pathway for energy expenditure
Source: EMBO J. 2014 Jan 15;33(5):418–36. doi: 10.1002/embj.201385014 (PMC3983686; doi:10.1002/embj.201385014)
Supplement: Supplementary file 5 [file embj0033-0418-sd5.pdf]

Book, Palm4,1.xls [Protected View] - Microsoft Excel

Row	A	B	C	E	G	H	I	J	K
5			1	1	1		1		1
6			MFN2,NE1U	MFN2,P.4,4:1	MFN2,P.2,4:1		MFN2,P.1,4:1		MFN2,P.05,4:1
7			Mean	Mean	Mean		Mean		Mean
8		EXP							
9	New algor	8.27.13, P1	95.51384208		116.8492093		49.93201923		34.30448068
10	New algor	8.27.13, P2	139.3651855		103.3627532		52.30397543		26.42915668
11	Old algory	Exp 3	85.2018857	176.7527818	87.12512692		29.91999564		14.57715201
12									
13		Average	106.6936378	176.7527818	102.4456965		44.05199677		25.10359646
14		SE	16.6047848		8.592846113		7.099099377		5.733227903
18			1	1	1		1		1
19			CONT,NE1U	CONT,P.4,4:1	CONT,P.2,4:1		CONT,P.1,4:1		CONT,P.05,4:1
20			Mean	Mean	Mean		Mean		Mean
21		EXP							
22	New algor	8.27.13, P1	100		98.39988494		44.04554053		24.92296471
23	New algor	8.27.13, P2	100		75.75984454		42.28650738		20.93223511
24	Old algory	Exp 3	100	132.4922666	50.11063514		24.97694928		14.81265961
25									
26		Average	100	132.4922666	74.7567882		37.10299906		20.22261981
27		SE	0		13.94892472		6.084251856		2.940081216
28									
29		pvalues	0.707480804	#DIV/0!	0.166277583	#	0.498619034	#	0.490898508

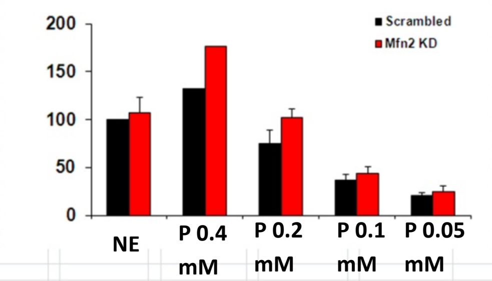

Sheet1 Sheet2 Sheet3

Original excel file data of the 3 independent seahorse experiments of the FFA titration on Mfn2KD/ Control.
