# Supplementary material for: Hormone-induced mitochondrial fission is utilized by brown adipocytes as an amplification pathway for energy expenditure
Source: EMBO J. 2014 Jan 15;33(5):418–36. doi: 10.1002/embj.201385014 (PMC3983686; doi:10.1002/embj.201385014)
Supplement: Supplementary file 6 [file embj0033-0418-sd6.pdf]

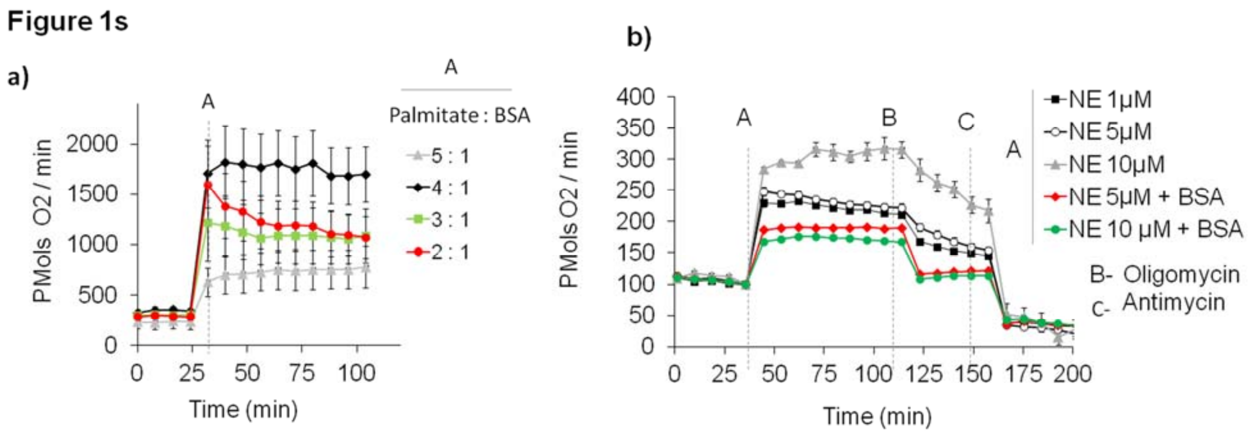

Supplementary Figure 1. Establishment of conditions that demonstate the synergistic effect of NE and FFA.

A) BSA concentration alters pa itate effect. Palmitate (0.4mM) was complexed to BSA in four different ratios (Palmitate:BSA; 5:1, 4:1, 3:1, 2:1) and added together with NE. Note that the Palmitate:BSA ratio used in the study was 4:1.

B) Norepinephrine titration. Not that the concentration of NE used in the study, 1µM, induce submaximal increase in oxygen consumption. In addition, adding BSA to NE inhibits the stimulated increase in oxygen consumption, indicating that intracellular concentrations of FFA are not saturating.
