# Supplementary material for: Hormone-induced mitochondrial fission is utilized by brown adipocytes as an amplification pathway for energy expenditure
Source: EMBO J. 2014 Jan 15;33(5):418–36. doi: 10.1002/embj.201385014 (PMC3983686; doi:10.1002/embj.201385014)
Supplement: Supplementary file 7 [file embj0033-0418-sd7.pdf]

Figure 2s

a)

b)

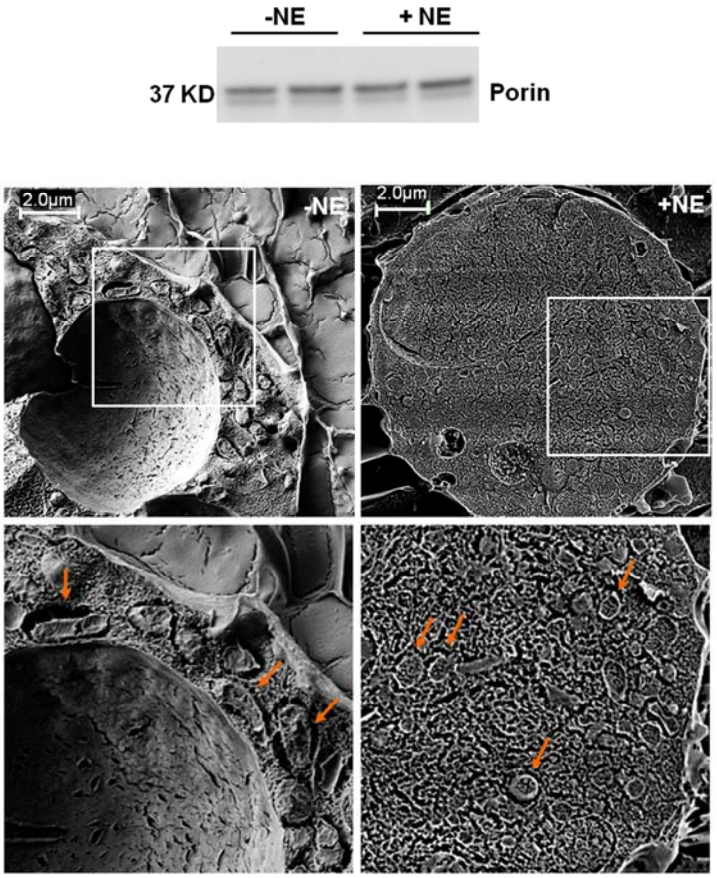

Supplementary Figure 2. Characterization of mitochondrial morphology and mass following BA stimulation with NE for 50 min in culture.

A) Mitochondrial mass, as measured by porin, does not change in response to NE treatment of BA in culture. Western blot of the mitochondrial marker Porin in the presence and absence of NE stimulation for 50 min. Note no change in mitochondrial mass after NE stimulation.

B) Scanning electron microscopy of mitochondrial fragmentation. Cultured brown adipocytes were stimulated by NE for 50 min followed by cryo-freezing and imaging. White square indicates zoom area. Arrows indicate mitochondria. Note the fragmented mitochondria in the NE treated cell. Scale bar 2 μm.
