# Supplementary material for: Hormone-induced mitochondrial fission is utilized by brown adipocytes as an amplification pathway for energy expenditure
Source: EMBO J. 2014 Jan 15;33(5):418–36. doi: 10.1002/embj.201385014 (PMC3983686; doi:10.1002/embj.201385014)
Supplement: Supplementary file 8 [file embj0033-0418-sd8.pdf]

Figure 3s

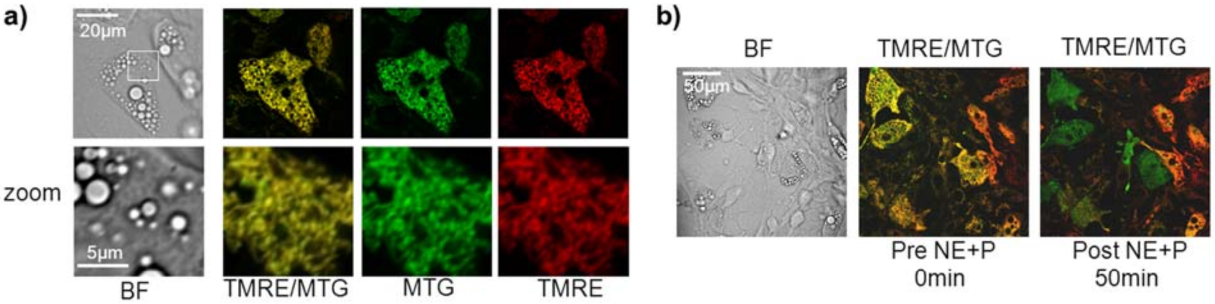

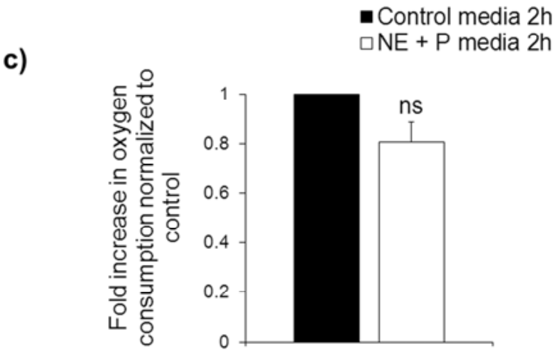

d) Table I: Recovery of mitochondrial morphology 24h after NE+P stimulation

	Cells with tubular mitochondri a (%)	Cells with fragmented mitochondria (%)	P-value
Ctrl (N=32)	75.5	23.9	
NE+P (N=26)	10.8	87.5	0.00001 (vs. both control and recovery)
Recover y (N=19)	67.4	31.3	0.29

Supplementary Figure 3. Recovery of mitochondrial morphology and $\Delta\psi_m$ after NE and palmitate stimulation. Cells were exposed to NE plus palmitate for a period of 2 h. NE plus palmitate were then removed and cells were incubated in normal culture media for additional 24 h before images were acquired.

A) Cell stained with TMRE/MTG under unstimulated conditions. Note the intact $\Delta\psi_m$. Scale bar 20µm, and 5µm for the zoom images.

B) Recovery of function 24 h after a 2 h stimulation with NE plus palmitate or control media; $\Delta\psi_m$ depolarization in response to NE and palmitate. Cells stained with TMRE/MTG. Scale bar 50µm.

C) Recovery of function 24 h after a 2 h stimulation with NE plus palmitate or control media; increase in oxygen consumption in response to NE. Data are normalized to the control cells response to NE (n=5 each condition).

D) Quantification of the recovery of mitochondrial morphology 24h after NE+Palmitate stimulation. Note that the mitochondrial fragmentation is recovered 24h after the NE+P was washed out.
