# Supplementary material for: Hormone-induced mitochondrial fission is utilized by brown adipocytes as an amplification pathway for energy expenditure
Source: EMBO J. 2014 Jan 15;33(5):418–36. doi: 10.1002/embj.201385014 (PMC3983686; doi:10.1002/embj.201385014)
Supplement: Supplementary file 9 [file embj0033-0418-sd9.pdf]

Figure 4s

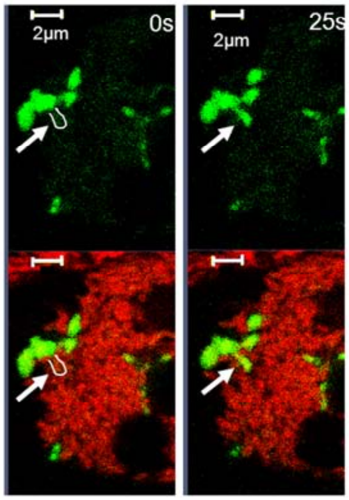

Supplementary Figure 4. Mitochondrial fusion event.

A) Cell expressing $_{mt}$DsRed and $_{mt}$PAGFP that was photoactivated prior to time-lapse imaging. Images are 3D projections from z-stacks to ensure that no mitochondrial segment moved out of the focal plane. Note the sudden increase in $_{mt}$PAGFP area at 25 s which represents a fusion event where $_{mt}$PAGFP is shared with another mitochondrion (white arrow). Scale bar 2µm.
