# Supplementary material for: Hormone-induced mitochondrial fission is utilized by brown adipocytes as an amplification pathway for energy expenditure
Source: EMBO J. 2014 Jan 15;33(5):418–36. doi: 10.1002/embj.201385014 (PMC3983686; doi:10.1002/embj.201385014)
Supplement: Supplementary file 10 [file embj0033-0418-sd10.pdf]

**Figure 5s**

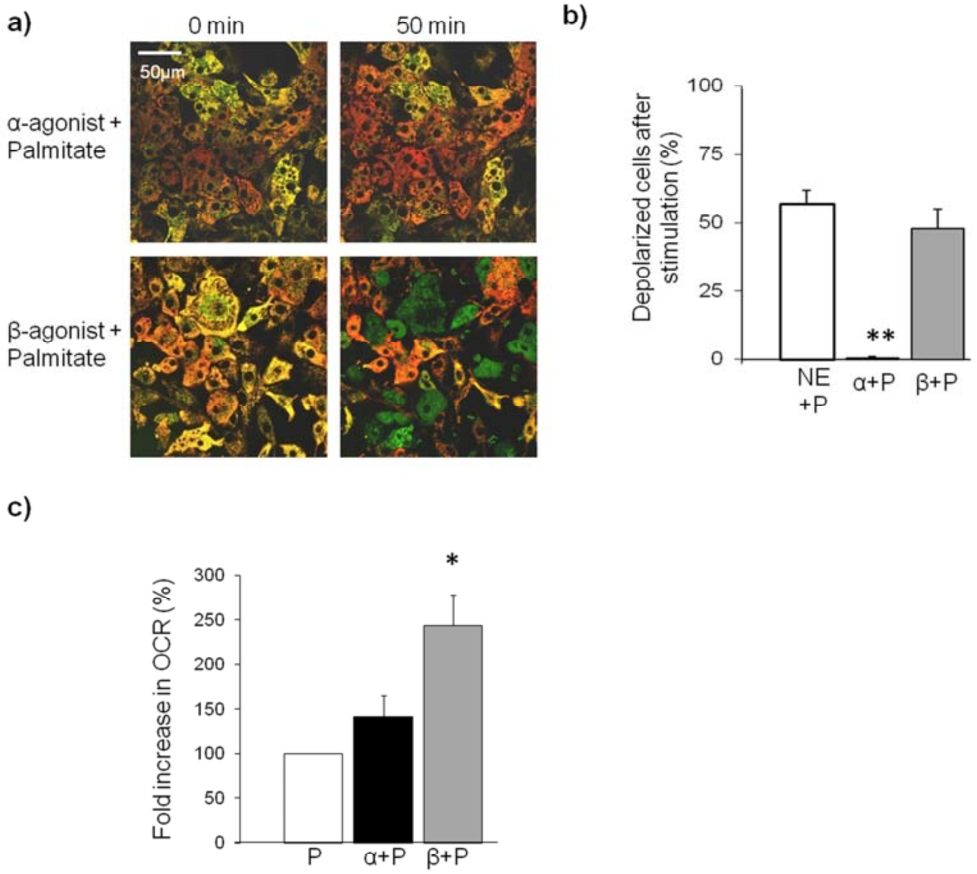


**Supplementary Figure 5. Synergistic effect on $\Delta\psi_m$ depolarization is mediated through β-adrenergic pathway.**

A) Images of multiple cells stained with TMRE/MTG and imaged 50 min after stimulation as indicated (only merged images are shown). Alpha-agonist = cirazoline (selective $\alpha_1$ agonist); Beta-agonist = CL-316243 (selective $\beta_3$ agonist). Cells that depolarize lose red TMRE staining and become green. Scale bar 50µm.

B) Quantification of the fraction of cells that undergo $\Delta\psi_m$ depolarization in response to stimuli as indicated. n=24 (NE + palmitate), n=10 (cirazoline + palmitate [α+P]), n=10 (CL-316243 + palmitate [β+P]). ** $p<0.01$. C) Synergistic increase in oxygen consumption by FFA plus NE involves the β-adrenergic pathway. Measurements of basal oxygen consumption rates were followed by measurements under palmitate -/+ CL-316243 or cirazoline. Note that data are normalized to Palmitate. N=4 (cirazoline [α+P]), n=3 (CL-316243 [β+P]). * $p<0.05$.
