# Supplementary material for: Hormone-induced mitochondrial fission is utilized by brown adipocytes as an amplification pathway for energy expenditure
Source: EMBO J. 2014 Jan 15;33(5):418–36. doi: 10.1002/embj.201385014 (PMC3983686; doi:10.1002/embj.201385014)
Supplement: Supplementary file 11 [file embj0033-0418-sd11.pdf]

Fig 6s

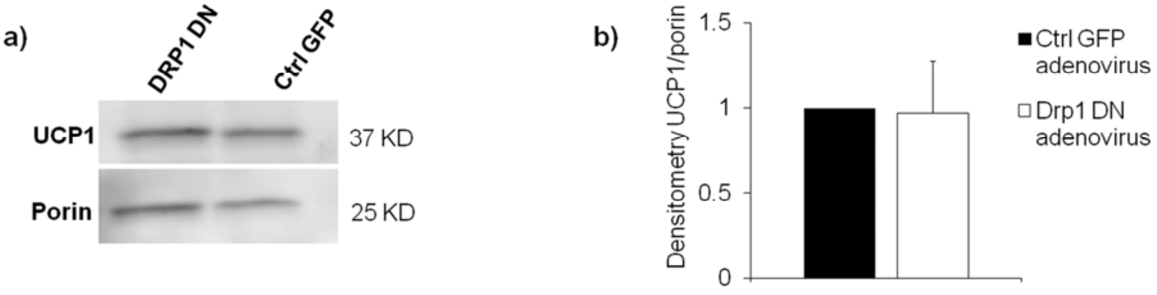

c) Table II: DRP1 DN effect on lipid droplets

n=22 cells / group	Ctrl	SE	DRP1 DN	SE	P-value
Lipid droplet count /cell	45.2	6.5	53.3	7.6	0.42
Average volume / lipid droplet (μm³)	24.3	6.0	18.9	3.7	0.45
Total lipid droplet volume / cell (μm³)	653.3	116.8	707.5	94.1	0.72

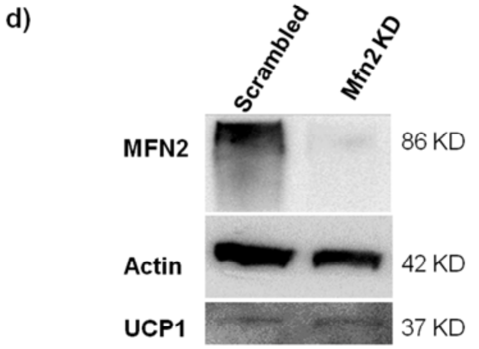

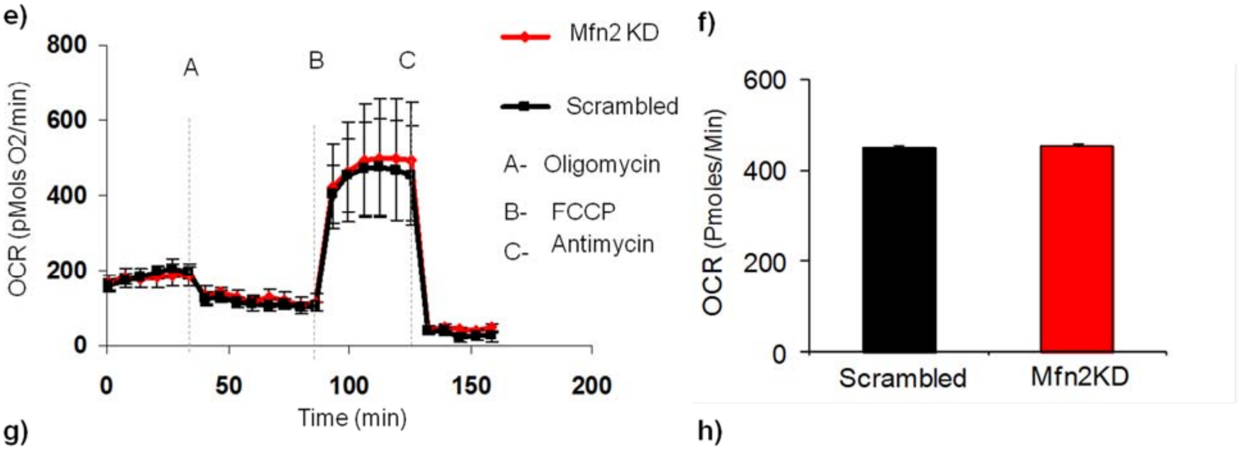

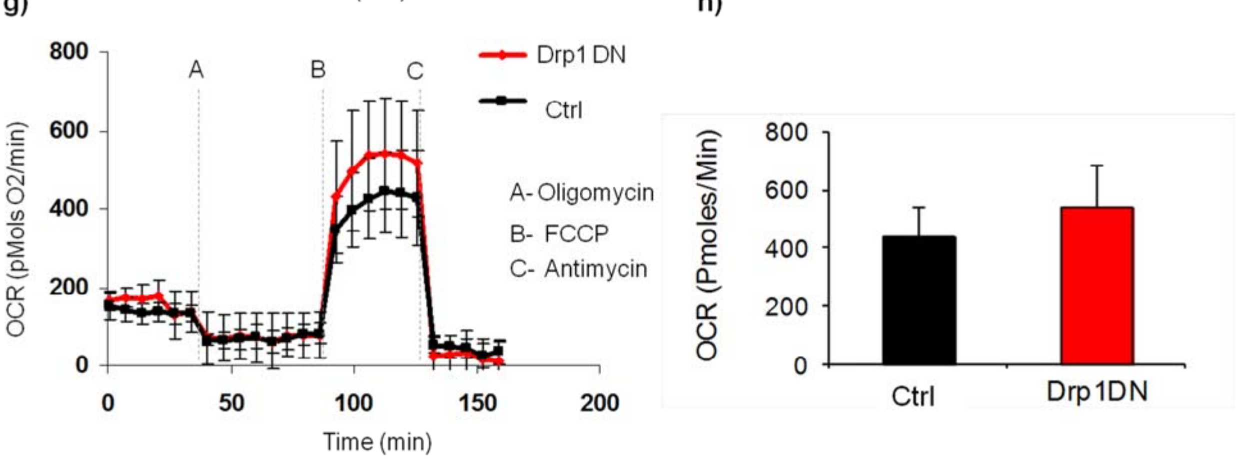

Supplementary Figure 6.

A-B) Western blot of Ucp1 content in cells expressing Drp1 DN vs. control. Porin as a loading control. Note that there is no significant difference in Ucp1 expression. n=5-6.

C) Effect of Drp1 DN on lipid droplet size and number as a marker of cell differentiation. Note that there is no significant difference between the control and Drp1 DN cells. N=22 cells per group.

D) Western blot of MFN2 knock down by microRNA and Ucp1 content. Actin as a loading control. Note the decrease in Mfn2 expression while Ucp1 expression was not affected.

E) Trace of oxygen consumption on Mfn2 KD and control cells after FCCP. Note that basal respiration and maximal capacity of mitochondrial oxygen consumption is not affected in the Mfn2 KD group. n=3 wells per group.

F) Quantification of the maximal oxygen consumption rate after FCCP injection of the control and Mfn2 KD groups. N=2.

G) Trace of oxygen consumption on Drp1 DN and control cells after FCCP. Note that basal respiration and maximal capacity of mitochondrial oxygen consumption is not affected in the Drp1 DN group. n=2 wells per group.

H) Quantification of the maximal oxygen consumption rate after FCCP injection of the control and Drp1 DN groups. n=2wells per group.
