# Supplementary material for: Hormone-induced mitochondrial fission is utilized by brown adipocytes as an amplification pathway for energy expenditure
Source: EMBO J. 2014 Jan 15;33(5):418–36. doi: 10.1002/embj.201385014 (PMC3983686; doi:10.1002/embj.201385014)
Supplement: Supplementary file 12 [file embj0033-0418-sd12.pdf]

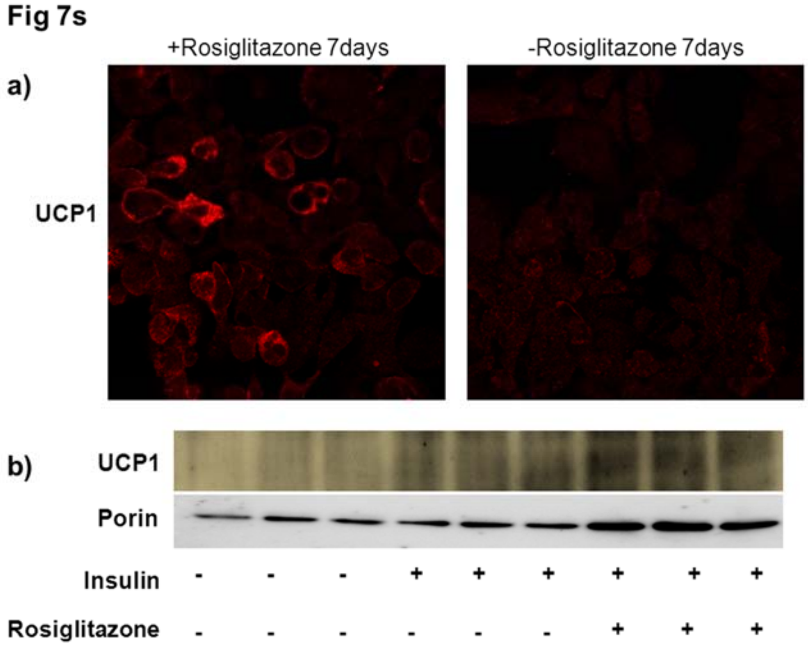

Supplementary Figure 7.

A) Ucp1 immunostaining of differentiated (with rosiglitazone) vs. undifferentiated cells (no rosiglitazone). Note the difference in fluorescence intensity.

B) Western blot for UCP1and porin in differentiated brown adipocytes in the presence and absence of insulin and rosiglitazone.
